# Antenatally Diagnosed Wilms’ Tumour

**Published:** 2014-01-01

**Authors:** YK Sarin, SK Rahul, S Sinha, N Khurana, S Ramji

**Affiliations:** Department of Pediatric Surgery, The Children’s Hospital and the Institute of Child Health, Lahore.; 1 Department of Pathology, Maulana Azad Medical College, New Delhi-110002, INDIA; 2 Department of Neonatology, Maulana Azad Medical College, New Delhi-110002, INDIA

**Keywords:** Wilms’ tumour, Hydrops foetalis, Antenatal detection

## Abstract

Wilms’ tumour (WT) is seldom seen in a neonate and prenatal diagnosis is rare. We present a case of antenatally diagnosed left sided WT with features of hydrops foetalis in a girl baby. Emergency LSCS was done at 34 weeks of gestation for foetal distress. Patient required mechanical ventilation for birth asphyxia and congestive cardiac failure. After stabilization, gross total resection of the tumour was done on day 4 of life. Histopathology HPE confirmed classical WT (stage I). Unfortunately, the patient died on the second postoperative day despite all supportive measures.

## INTRODUCTION

Neonatal Wilms’ tumour (WT) is rare; the most common neonatal renal tumour being Congenital Mesoblastic Nephroma (CMN/ Congenital renal hamartoma) comprising 66.7% of all neonatal renal masses [1, 2]. Only 0.12% of all Wilms’ tumours in NWTS 1-3 study were less than 30 days old vis-a-vis 0.54% CMN [2]. We came across an antenatally diagnosed WT, the details of whom are described here. 

## CASE REPORT

A 20-year-old second gravida mother was referred to us at 29+5 weeks of gestation for prenatal ultrasonography (USG) findings of polyhydramnios associated with a heterogeneous left renal mass measuring 3.5 x 5.5 x 4.7 cms with solid and cystic components. She had had a previous LSCS at 28 weeks for pre-eclampsia with placenta abruption. During the current pregnancy, she had malaria in the first trimester and varicella in the second trimester. Scans during early pregnancy did not reveal any anomaly. Serial USG done 4 weeks later showed increase in size of the tumour to 9.8 x 8.7 cms along with features of hydrops foetalis (thickened foetal skin, scalp oedema, foetal ascites and cardiomegaly), polyhydramnios and foetal distress. Hence, emergency LSCS was done at 34 weeks and a 3420 G girl baby was born.

The newborn required resuscitation for poor APGAR scores which was followed by mechanical ventilation. On examination, there was generalised anasarca, anaemia and a large retroperitoneal tumour on the left side. There was no haematuria or hypertension. Postnatal USG confirmed the large left renal mass crossing the midline associated with ascites. Ascitic tap revealed transudative fluid. Laboratory investigations showed anaemia, leucocytosis and thromcytopenia. Peripheral smear showed the presence of some nucleated red blood cells. She required platelet transfusion for progressive decrease in platelet counts. After optimising the child, guided fine needle aspiration cytology (FNAC) was done which showed evidence of small round blue cell tumour. Following this, left nephroureterectomy was done on day 4 of life (Fig. 1). Gross total resection of a 12x10 cm left renal tumour was done without any tumour rupture/spillage. The opposite kidney was normal. The child was kept on ventilator in the neonatal intensive care unit. Postoperatively, the condition of the baby deteriorated despite all possible measures and she died on the second postoperative day. Histopathology (HPE) (Fig. 2) report showed evidence of Wilms’ tumour with areas of focal necrosis, haemorrhage, cystic changes and capsular infiltration without any breach; perinephric fat was free of tumour. Renal pelvis, hilum and ureter were free of the tumour. 

**Figure:**
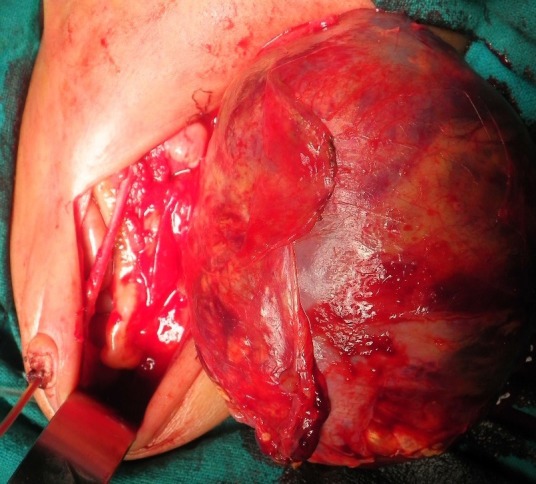
Figure 1: Intraoperative picture showing large Wilms’ tumor.

**Figure:**
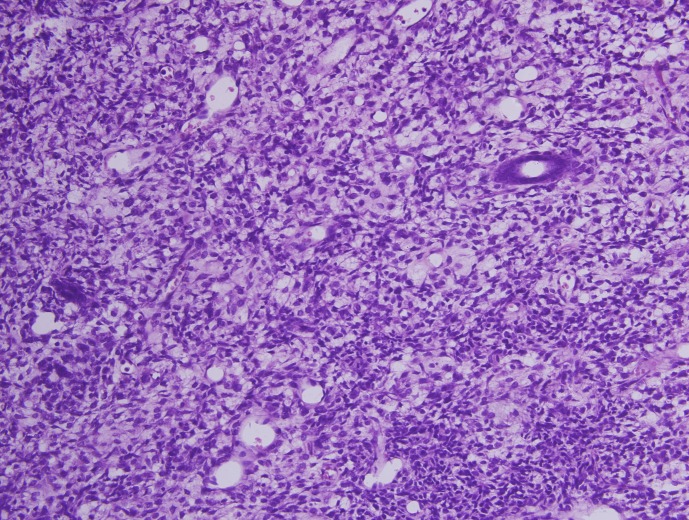
Figure 2: Histopathology showing classical Wilms’ tumor

## DISCUSSION

Antenatally diagnosed solid renal masses could be CMN, suprarenal neuroblastoma, adrenal haemorrhage, Wilms’ tumour or retroperitoneal teratoma; of which CMN is the commonest [3, 4]. Prenatal detection of Wilms’ tumour is extremely rare [5, 6]. Besides ultrasonography, foetal magnetic resonance imaging (MRI) has been found to be helpful in resolving the diagnostic dilemma of foetal renal masses [7-10]. Imaging guided FNAC, when feasible can confirm the diagnosis [4]. Unfortunately, there isn’t much scope of prenatal intervention for foetal renal tumours and definitive intervention is usually deferred until birth.

An antenatally detected renal tumour may be associated with polyhydramnios, hydrops foetalis and acute foetal distress [4]; of which polyhydramnios is the commonest while hydrops is rare. Our patient had evidence of foetal hydrops and deranged coagulation parameters. The rapidly growing tumour (which almost doubled in size in 4 weeks’ time) could be held responsible for the hydrops; due to compression of the infradiaphragmatic great vessels. In addition, vascular shunting into the tumour could also have led to circulatory failure and hydrops. Worsening of hydrops and rapid increase in the tumour size on serial USG made us suspicious about the malignant nature of the mass. As was seen in our case, foetal hydrops, when present, is a poor prognostic factor and often leads to demise of the newborn.

The management of this patient posed a major challenge. Prematurity, worsening hydrops, haemodynamic instability, cardiomegaly and the malignancy per se made the survival chances dismal. Early surgery after optimum stabilization was undertaken in an attempt to improve the circulatory physiology by reducing the tumour burden, though in vain. As per the HPE and staging, it was a localized WT with good prognosis. Associated hydrops and polyhydramnios worsen the prognosis.

Had she survived, her further management would again have been a matter of concern. There are no clear guidelines on the use of adjunctive therapy in neonates and infants. Radiotherapy does not have documented safety in infants and chemotherapy in regular doses is toxic and poorly tolerated. In our patient, since the tumour weight was less than 550 gram and HPE was favourable, we would not have given any further chemotherapy and would have kept her on close follow-up instead.

Although neonatal WT usually has a good prognosis, close follow-up is essential as adjuvant therapy is often impractical and recurrence is likely to cause further morbidity and mortality. In a resource challenged nation like ours, foetal diagnostic tests are far from accurate, there is a lack of definite algorithm for antenatal management and foetal surgery is still in its early days.

## Footnotes

**Source of Support:** Nil

**Conflict of Interest:** None

